# Characterization of sensitivity encoded silicon photomultiplier (SeSP) with 1-dimensional and 2-dimensional encoding for high resolution PET/MR

**DOI:** 10.1186/2197-7364-1-S1-A83

**Published:** 2014-07-29

**Authors:** Negar Omidvari, Volkmar Schulz

**Affiliations:** Philips Technologie GmbH Innovative Technologies, Research Laboratories, Aachen, Germany; RWTH Aachen University, Aachen, Germany; Department of Physics of Molecular Imaging Systems, Institute for Experimental Molecular Imaging, RWTH Aachen University, Aachen, Germany

Semiconductor based photo detectors such as avalanche photo diodes (APDs) and silicon photomultipliers (SiPMs) in combination with fast scintillator crystal arrays have shown to be promising detector technologies for simultaneous PET/MR. Sub-mm spatial resolution can be achieved by one-to-one coupling of APDs and SiPMs to small pitched crystal arrays. However, this results in a high number of readout channels and electronics, resulting in increased power consumption. This paper evaluates the performance of a new type of PET detectors called sensitivity encoded silicon photomultiplier (SeSP), which allows a direct coupling of small pitch crystal arrays to the detector with a reduction in number of readout channels. Four SeSP devices have been investigated in this study, which were designed with two separate encoding schemes: 1D and 2D. Furthermore, both encoding schemes were manufactured in two different sizes of 4×4 mm^2^ and 7.73×7.9 mm^2^ in order to investigate the effect of size on detector parameters. All devices were coupled to LYSO crystal arrays with 1 mm pitch size and 10 mm height, with optical isolation between crystals. The characterization has been done for the key parameters of crystal-identification, energy resolution, time resolution, and dark noise ratio as a function of triggering threshold, over-voltage (OV), and temperature. Position information has been archived using a least square approach (LSQA) in combination with a mean light matrix around the photo-peak. The positioning results have proved the capability of all four SeSP devices in precisely identifying of all crystals coupled to the sensors. The separation of crystals was better in lower bias voltages in all four devices. Energy resolution was measured for the devices in different measurements varying from 12 to 18% (FWHM) and paired coincidence time resolution (pCTR) of 400 ps to 1.1 ns was obtained for different SeSP devices at room temperature.

